# The conformational state of the nucleosome entry–exit site modulates TATA box-specific TBP binding

**DOI:** 10.1093/nar/gku423

**Published:** 2014-05-14

**Authors:** Aaron R. Hieb, Alexander Gansen, Vera Böhm, Jörg Langowski

**Affiliations:** Division Biophysics of Macromolecules, German Cancer Research Center, D-69120 Heidelberg, Germany

## Abstract

The TATA binding protein (TBP) is a critical transcription factor used for nucleating assembly of the RNA polymerase II machinery. TBP binds TATA box elements with high affinity and kinetic stability and *in vivo* is correlated with high levels of transcription activation. However, since most promoters use less stable TATA-less or TATA-like elements, while also competing with nucleosome occupancy, further mechanistic insight into TBP's DNA binding properties and ability to access chromatin is needed. Using bulk and single-molecule FRET, we find that TBP binds a minimal consensus TATA box as a two-state equilibrium process, showing no evidence for intermediate states. However, upon addition of flanking DNA sequence, we observe non-specific cooperative binding to multiple DNA sites that compete for TATA-box specificity. Thus, we conclude that TBP binding is defined by a branched pathway, wherein TBP initially binds with little sequence specificity and is thermodynamically positioned by its kinetic stability to the TATA box. Furthermore, we observed the real-time access of TBP binding to TATA box DNA located within the DNA entry–exit site of the nucleosome. From these data, we determined salt-dependent changes in the nucleosome conformation regulate TBP's access to the TATA box, where access is highly constrained under physiological conditions, but is alleviated by histone acetylation and TFIIA.

## INTRODUCTION

Gene-specific transcription is complex, requiring the concerted effort of many factors to recognize specific DNA loci and make them accessible to the transcription machinery. This process requires at least the ordered assembly of the general transcription machinery (TATA binding protein (TBP), TFIIB, TFIIA, TFIIF and RNA polymerase II) to the promoter, forming the preinitiation complex (PIC). Assembly is initiated by the sequence-specific association of TBP to the promoter (1,2). Furthermore, assembly occurs in the context of chromatin, which limits access to the genomic DNA and acts as a key regulator of PIC formation. The majority of this regulation occurs around the nucleosome, the basic repeating unit of chromatin. The nucleosome wraps 147 bp of DNA ∼1.7 times around an octameric protein core, consisting of two copies each of histones H2A, H2B, H3 and H4 (3). Evidence is growing that the nucleosome is highly dynamic and modular (4–6), which defines a competition between nucleosome and transcription factor occupancy (7).

Since TBP is central to PIC assembly, a rich body of research exists characterizing TBP's association to promoter DNA. Briefly, TBP is a minor groove DNA binding protein that can bind nearly any DNA sequence, but has a preference for the TATAWAWR (TATA box) consensus motif (8,9), typically associated 25–30 bp upstream of the transcription start-site (tss). TBP binds the TATA box with high affinity (*K_d_* ≈ 2 nM) by inducing a bend of ∼80° in yeast or 105° in human (10–13). An increase in the average bend angle is generally correlated with stronger binding affinity *in vitro* and enhanced transcriptional activity *in vivo* (10,13,14). On the other hand, recent single-molecule data has shown no significant differences in bend angle between sequences, and previous differences observed under bulk conditions may simply reflect their apparent binding affinities. From these data, the authors postulated two possible models for TBP binding to TATA box DNA. The first follows a linear pathway (Reaction Scheme 1A) with an intermediate containing a conformational state undifferentiated from the final bound/bent state, but with differing kinetic lifetimes. The second follows a branched model (Reaction Scheme 1B), wherein TBP can associate as two distinctly different species with comparable bend angle but with different thermodynamic stabilities.

**Figure:**
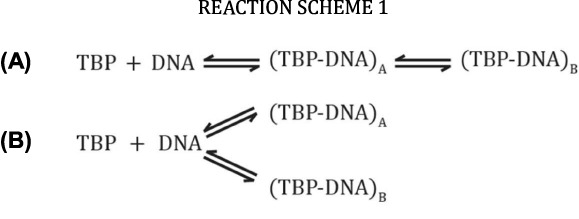


In addition to the sequences within the TATA box, Faiger *et al.* have shown that DNA sequences flanking the TATA box can influence the thermodynamic and kinetic properties of the TBP-DNA complex (8,15,16). Furthermore, specificity and affinity of TBP toward the TATA box can be influenced by factors, such as TFIIA, TFIIB, Mot1 or NC2 (17–19). Therefore, insight into the sequence-dependent effects defining TBP binding and understanding how external factors affect this process will help us to refine the mechanisms of sequence-specific TBP-promoter binding.

Within chromatin, TATA box containing promoters directly compete between TBP binding and nucleosome occupancy, however, only relatively few genes contain TATA box motifs (<15% in yeast and <10% in human) (20,21). At these non-consensus promoters, PIC assembly depends on TFIID and is directly correlated with positioning of a nucleosome downstream of the tss (20,21). How TBP differentiates between consensus or non-consensus sites during ordered PIC assembly is not understood (22). TBP binding to TATA box elements buried within the nucleosome requires adenosine triphosphate-dependent remodeling, which exemplifies the role of the nucleosome in regulating PIC assembly (23). *In vivo*, this process is biphasic: first the nucleosome is moved downstream to accommodate TBP binding, followed by additional movement to facilitate complete PIC assembly (24). Additionally, high-resolution ChIP-seq data show a positioned nucleosome covering the tss on TATA box containing promoters that correlates with TATA-box position and phasing within the nucleosome footprint (25). This suggests the nucleosome may be judiciously placed relative to the TATA box to obtain specific function; however, it is not clear whether complete displacement or architectural changes within the nucleosome are required for initial TBP recruitment.

The nucleosome, long thought to be a stable complex, has been shown to be a highly dynamic and modular system by a number of biophysical studies. In particular, the DNA entry–exit site of the nucleosome shows transient exposure to the exiting DNA and marks the most accessible point within the nucleosome, which may be specifically targeted for TBP (or other transcription factor) binding (23,26,27). This is highlighted by work from the Widom and Poirier labs who have shown that the entry–exit site dictates sequence-specific access to the major groove binding protein LexA by acting as a competitive inhibitor to sequence-specific binding (28,29). Additionally, deletion of the histone tails or addition of histone modifications, specifically acetylation, increases TBP accessibility and is correlated with enhanced transcriptional activity (27,30), while the phasing and depth of the TATA box within the nucleosome affect TBP accessibility and may play an important role in transcription regulation (25). These data bring forward an interesting mechanistic model, suggesting the position of the TBP binding-site within the nucleosome and the architecture of the nucleosome play important roles in regulating PIC assembly. Understanding how the nucleosome becomes positioned at particular locations and the means by which it is made structurally accessible are outstanding questions that require further mechanistic study.

Using bulk and single-molecule FRET experiments, we characterized TBP binding to DNA and observed the discreet states formed during TBP association to the TATA box. TBP binding to a short minimal TATA box fragment, at equilibrium, is compatible with a two-state binding model with no discernible intermediate conformations. In contrast, we detect multiple DNA conformations when flanking DNA sequence is added to the TATA box, indicating increased non-specific association to the DNA. These non-specific interactions become most prominent at elevated TBP concentrations indicating a cooperative binding effect, and can be alleviated by increasing monovalent salt concentration, the addition of TFIIA or competitor DNA. Furthermore, kinetic experiments show at least two distinct binding states that are pronounced upon initial binding, but are directed toward the most stable kinetic state at equilibrium. Additionally, TBP's canonical binding is highly suppressed by a nucleosome occupied TATA box in a salt-dependent manner, forcing TBP to bind either off-consensus or in an alternative architecture under physiological conditions. Binding is significantly de-repressed in the presence of acetylated histones or the addition of TFIIA, showing two synergistic methods by which TBP recruitment can be enhanced at nucleosome occupied DNA. These data give us a revised model for TBP association to DNA and shows how nucleosome architecture can be modulated to regulate PIC assembly.

## MATERIALS AND METHODS

### Protein purification

Full-length human TBP was expressed and purified as previously described (5), with an additional MonoQ column used for a final purification step by collecting the flow through containing TBP. TFIIA subunits α/β and γ were expressed and purified as previously described (10).

### DNA constructs

DNA oligonucleotides (Supplementary Figure S1) were fluorescently labeled by conjugating the specified succinimidyl ester dye to a C6 5′ or C6 amino dT linker for internally labeling, as previously described (10). Depending upon the construct, Atto-488 was used as donor dye, while Atto-633 or Atto-647N was used as acceptor dye, with an estimated Förster radius (*R*_0_) of ∼51 Å for each pair. Labeled oligonucleotides were subsequently purified by reverse-phase high pressure liquid chromatography. TATA-601 DNA (Supplementary Figure S7) is a 159 bp DNA construct in which the TATA box was inserted into the ‘601’ Widom sequence (31), ∼5 bp from the last histone-DNA contacts. TATA-601 DNA was synthesized through PCR using the TATA-14I primer and purified via gel electrophoresis.

### Nucleosome preparation

Histones and nucleosomes were prepared as previously described (32). Briefly, nucleosomes were assembled through salt-dialysis by combining TATA-601 DNA with histone octamer. Recombinant histones were chemically acetylated by incubating them in 0.1 M acetyl phosphate for 3 h at 50 °C. Endogenous histone octamers were isolated from HeLa cells collected under ‘normal’ conditions, or hyperacetylated by soaking the cells in 6 mM sodium butyrate for 6 h (32).

### Microplate-based FRET binding experiments

Binding experiments between TBP and fluorescently labeled DNA were performed in 384-well glass bottom microplates (Sensoplate Plus, Greiner BioOne). Microplates were cleaned and passivated with SigmaCote™, as previously described (33). Labeled DNA was diluted to a concentration 2-fold over the final concentration (1 nM) in binding buffer containing 20 mM Tris (pH 7.9), 4 mM MgCl_2_, 1 mM DTT, 5% glycerol and KCl as indicated. Note that 15 μl of 2× DNA was then added to each well of the microplate. A total of 15 μl of 2× TBP titration points were then added to the DNA in the microplate. Microplates were then shaken on an MS2 Minishaker (IKA works) for 30–60 s. Reactions were allowed to incubate for 20 min and then scanned on a Typhoon 9400 variable mode imager (GE Healthcare). Microplates were elevated, to prevent imaging of the meniscus, by suspending the microplate on two 1 mm glass slides stacked on each side. Microplates were scanned with a pixel resolution of 100 μm, focus position +3 mm and press ‘on’; a total of three images corresponding to Donor, Acceptor and FRET signals were taken as indicated below. Fluorescence excitation and emission settings are detailed in the ‘Calculation of Proximity Ratio’ section of Supplementary Materials and Methods. Images were quantified using ImageQuant software. From the three fluorescence channels, FRET signal was corrected for background, crosstalk and direct excitation, with proximity ratio calculated as described in Supplementary Materials and Methods. Quantified data were then plotted and approximated by the following hyperbolic function representing single-site binding, using Prism (GraphPad) software:

(1) \begin{equation*} P = P_{\min } + (P_{\max } - P_{\min } ) \cdot \left( {\frac{{[T]}}{{[T] + K_D }}} \right) \end{equation*}

where *P* is the proximity ratio and [*T*] is the total concentration of TBP added.

### spFRET experiments

spFRET experiments were performed on a home-built confocal microscope, as previously described (34). Reactions were performed in SigmaCote™ treated 384-well microplates, as described above in 40 μl total reaction volume. DNA was diluted to 30–70 pM and subsequently equilibrated with the specified TBP concentrations in binding buffer (0.5 mM MgCl_2_) containing the indicated KCl concentration for 20 min; samples additionally contained 0.5–1 mM ascorbic acid to minimize photobleaching. Samples were illuminated with 488 nm laser light with donor (535AF45 filter) and acceptor (HQ705/90 filter) emission signals collected on avalanche photodiodes (SPCM-AQ-14, PerkinElmer) for 30 min. Single photon data from a time-correlated-single-photon-counting board (TimeHarp200, PicoQuant) were analyzed using in-house software; single-molecule events were discriminated against background following a burst selection protocol that is described elsewhere (34). Proximity ratios and histograms were then generated in IGOR software (Wave Metrics) with data fit using the integrated Gaussian fitting function; Gaussian curve-fits in Figure 2 excluded the donor-only peak.

**Figure 1.**
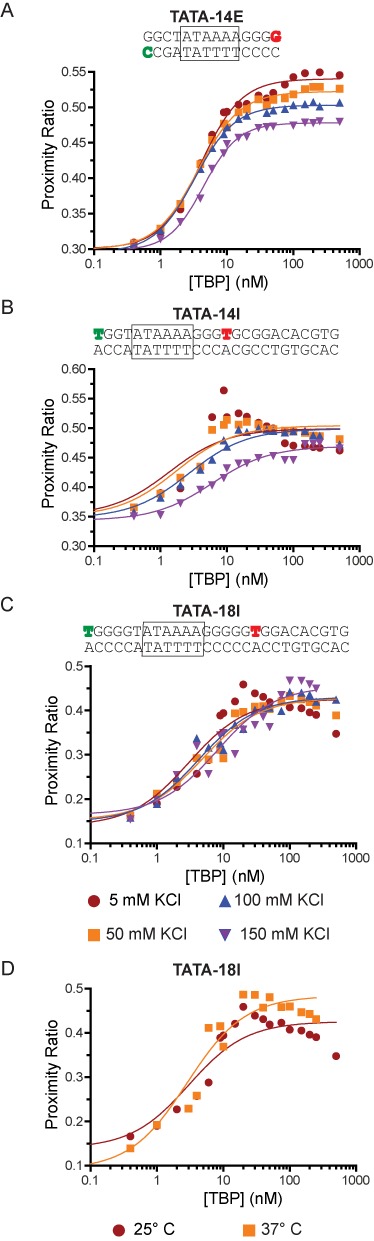
TBP binding to TATA box DNA is altered in the presence of adjacent DNA. Testing of TBP binding to (**A**) TATA-14E, (**B**) TATA-14I and (**C**) TATA-18I DNA constructs at varying salt concentrations. DNA sequences and labeling positions are shown in above their respective figure (green-donor, red-acceptor). The plots show binding isotherms for increasing amounts of TBP, where an increase in proximity ratio is due to TBP-induced DNA bending. (**D**) Comparison of binding to the TATA-18I construct at 25°C and 37°C. All data are fit to a single-site binding isotherm using Equation (1). In many cases (B–D) the data do not fit this model, but are in place to highlight the deviation from normal binding behavior. Supplementary Figure S2 shows (A) and (B) as globally fit data.

**Figure 2.**
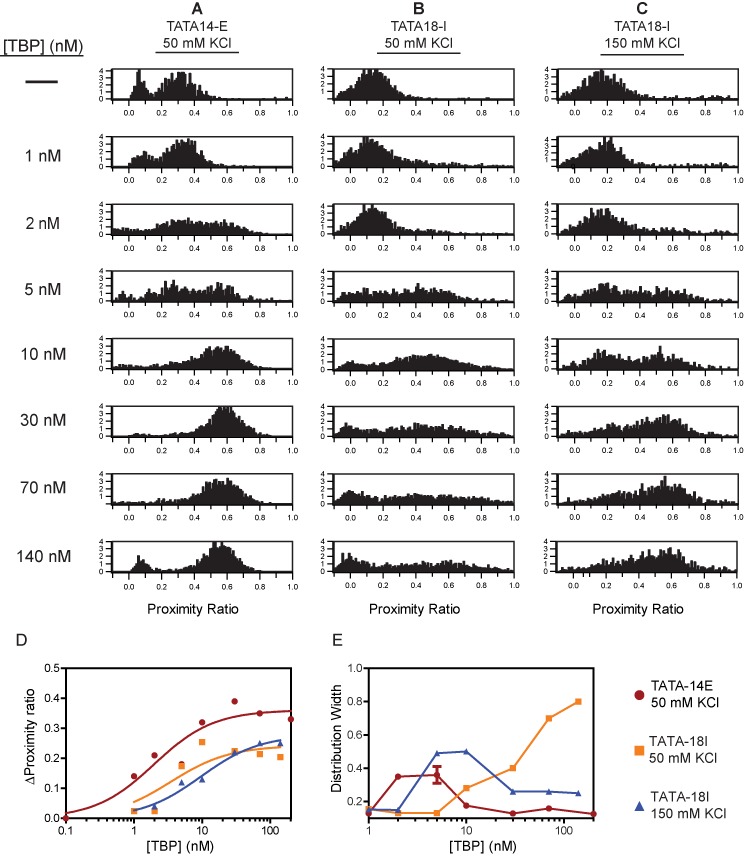
spFRET analysis reveals length-dependent increases in DNA bending heterogeneity at saturating TBP concentrations. (**A**) Proximity ratio distributions obtained using spFRET on TATA-14E construct at 50 mM KCl, with varying concentrations of TBP. *Y*-axis′ are shown as relative frequencies of each FRET state. (**B–C**) spFRET histograms obtained on TATA-18I DNA at 50 (B) and 150 (C) mM KCl. (**D**and **E**) Plots showing the change in (D) average proximity ratio and (E) the distribution width for each histogram in A–C. Data in (D) are fit to Equation (1). Average proximity ratios were calculated as an average of the whole population. Bound peak proximity ratios represent the proximity ratio of all particles measured. Distribution widths were obtained by fitting the corresponding histograms to a single Gaussian, excluding the donor-only peak. Data in (E) are connected by a line for easier viewing and do not represent fitting of data. For a normal two-state binding system, the distribution would first rise and then fall after reaching 50% binding and plateau to approximately the same value of the unbound DNA.

### Kinetic experiments

Kinetic experiments were performed on the same confocal microscope used for spFRET. Briefly, labeled DNA (1 nM), was incubated with 30 nM TBP in binding buffer containing 50 mM KCl, in a treated 384-well microplate. After the specified incubation times, unlabeled DNA (2 μM) was added to the reaction and mixed by pipette. Donor and FRET intensities where then collected over time. The proximity ratio was calculated, for equal length time bins, and plotted versus time in Prism software. Data were fit to a double exponential decay curve, included in the software.

## RESULTS

### Flanking DNA sequence length alters the TBP induced TATA box conformation

In the cell, TBP encounters DNA in a variety of chromatin states and is recruited to a broad set of sequences. Therefore, to understand how TBP associates with specific sites in the genome, detailed studies of TBP association to these states must be performed. In particular, we were interested in how TBP interactions compare between naked and nucleosome occupied DNA; TBP encounters each of these scenarios readily within the nucleus. Therefore, to gain a better grasp of TBP binding properties in the presence of extended DNA, we compared TBP binding to a minimal TATA-box and one with flanking DNA, in real time using FRET. To measure TBP binding to DNA, we used the FRET pair-labeled DNA fragments, shown in Figure 1 and Supplementary Figure S1, and measured the extent of TBP-dependent bending of the DNA. As TBP binds the TATA box, it bends the DNA, bringing the two fluorophores closer together, thus increasing the FRET signal, or proximity ratio (Supplementary Figure S1E); this method is a proven read-out for TBP-DNA association (10,35–37). Equilibrium binding experiments were performed in 384-well microplates by titrating TBP onto a constant concentration of labeled DNA (1–5 nM), with proximity ratios calculated (Supplementary Equations (1)–(4)) following imaging of the microplate on a Typhoon variable mode imager.

We initially tested the previously characterized TATA-14E construct, a 14 bp DNA with its 5′ ends containing donor and acceptor pairs (Figure 1A); globally fit data are shown in Supplementary Figure S2. With this substrate, we observe an increase in proximity ratio as TBP concentration increases, showing that TBP binds DNA tightly, *K_d_* = 5 ± 1 nM at 50 mM KCl. This is comparable with previous experiments showing 2–5 nM binding affinity under similar conditions (10,12). Notably, our TBP preparation shows a high level of functional activity, as seen from stoichiometry measurements (Supplementary Figure S3). Since a variety of conflicting results have accumulated regarding TBP binding (8,12,37,38), we asked whether this may be due to salt-dependent differences in experimental conditions. Therefore, we compared TBP binding to the TATA box under varying salt conditions from 5 to 150 mM KCl. From these data, we find little difference between KCl concentrations, other than slight salt-dependent decreases in apparent binding affinity and proximity ratio at saturation; this reduction in proximity ratio could be reflected as a slight change in bend angle or conformation when bound. We do not see a strong dependence on binding affinity until 150 mM KCl, suggestive that the binding affinities at lower ionic strength are maximized in stability or that our probe concentrations are greater than or near the apparent binding affinity, due to sensitivity limitations.

To understand how additional DNA adjacent to the TATA box affects this interaction, we tested binding of TBP to TATA-14I, a 25 bp DNA containing the TATA box positioned near one end of the DNA and an additional 11 bp added on the side flanking the TATA box (Figure 1B), relative to TATA-14E. Since FRET experiments are limited to distances less than about twice the Förster radius, and to be consistent with the previous experiment, we placed the donor fluorophore on the 5′ end of the DNA and the acceptor fluorophore internally on the same strand, 14 bp away. Strikingly, binding of TBP to this construct at KCl concentrations less than 100 mM leads to two-phase binding isotherms compared to the minimal TATA-14E. This curve is characterized by an initial hyperbolic shape through the linear phase of binding, but decreases sharply at elevated TBP concentrations, plateauing at a significantly lower value. The sharpness of the decrease is strongest at 5 mM KCl and disappears by 150 mM KCl. The data are approximated by a hyperbolic binding isotherm, Equation (1), to highlight their deviation from normal binding behavior. The affinity at 150 mM KCl (6 ± 1 nM) is comparable to that observed on TATA-14E (10 ± 3 nM).

To exclude the possibility that the internal label affects binding, we changed the acceptor label position, such that it was 18 bp from the donor fluorophore (TATA-18I; Figure 1C). When binding TBP onto TATA-18I, we observe an increased change in proximity ratio but the same overall binding pattern, indicating the fluorophore position is not responsible for this binding behavior. Additionally, since TATA-14E differs slightly in sequence from TATA-14I, we also probed TATA-14E* (sequence shown in Supplementary Figure S1B), giving similar results to those for TATA-14E (data not shown); this suggests the difference in sequence is not responsible for the biphasic behavior. We also found no significant changes in fluorophore anisotropy upon binding to either construct (data not shown). Considering the binding behavior of TBP to DNA can be influenced by salt concentration, we tested whether temperature could also alter these binding state(s). Figure 1D shows that binding performed at 37°C does not significantly alter the TBP binding behavior, in line with previous experiments showing TBP binding is not significantly influenced by temperature (39). This indicates that entropic, not enthalpic, forces are driving TBP binding (40,41). Together, these data suggest that reduction in ionic strength leads to alternative binding behavior on long DNA fragments.

### spFRET reveals alternative binding-site positioning of TBP to TATA box DNA

As we have shown, the addition of DNA flanking to the TATA box strongly influences the binding properties of the TBP-DNA complex. However, to elucidate the precise conformational states for each DNA, more powerful techniques are needed. For example, previous studies suggested that two or more different intermediate states are present during binding of TBP to a minimal TATA-DNA (13,16), while more recent spFRET studies on human TBP indicate a two-state system (37,42). To test whether flanking DNA influences the formation of any intermediate states, we used single-pair FRET (spFRET) spectroscopy (43) to resolve the specific DNA conformations induced by TBP binding. The confocal microscope system used here allows molecules to diffuse freely, eliminating potential artifacts from surface immobilization. Single-molecule events were detected by diluting the DNA to a concentration (<100 pM) at which most particles diffuse individually through the confocal volume, resulting in well-separated bursts of signal in both donor and acceptor emission channels upon donor excitation. These individual events are then quantified and plotted as a histogram of proximity ratios.

We initially probed TATA-14E using varying TBP concentrations in 50 mM KCl (the salt concentration typically used for *in vitro* transcription) to test TBP binding under the most basic conditions (44). If multiple conformational states are formed upon TBP binding, as suggested on such short DNA fragments, we would expect it to be most clearly visible on this construct. Histograms from these experiments are shown in Figure 2A, where DNA alone is characterized by two peaks, one centered around a proximity ratio near 0.0 and another at 0.3, each representing DNA containing donor-only (missing or inactive acceptor) or donor-acceptor pair (DNA_unbound_), respectively. As TBP is gradually increased in concentration, the DNA_unbound_ peak decreases in height, while another peak near 0.6 arises, signifying DNA is in the bound/bent state (DNA_bound_). The proximity ratios from bulk (at saturation) and spFRET are similar, but not the same, because data were not corrected for gamma (γ); a normalization factor reflecting the relative detection efficiencies in the donor and acceptor channels, as well as differences in quantum yields (45). Interestingly, in these data, we do not observe any intermediate states, even at subsaturating TBP concentrations, where a buildup of intermediates would most likely form. However, due to resolution limitations, we cannot completely exclude trace amounts of intermediates or those which could be hidden within peaks. To compare this data to our bulk data, we calculated average proximity ratios for complete spFRET data sets, as well as fit the data to a single Gaussian, and plotted average bulk proximity ratios and the width of the distribution for each TBP concentration. Average proximity ratio is plotted in Figure 2D, showing a concentration-dependent binding isotherm analogous to those seen in Figure 1A. Thus, these data indicate that TBP bound to TATA-14E DNA, at equilibrium, is in a two-state system, with no discernible intermediate states.

To resolve the curious biphasic binding behavior in Figure 1, we next probed TBP binding to TATA-18I DNA at 50 mM KCl. We chose TATA-18I over TATA-14I due to the larger difference in proximity ratios between unbound and bound states, giving greater resolution and increased opportunity to observe multiple conformational states. Figure 2B shows the spFRET histograms on TATA-18I under increasing TBP concentrations. Upon binding with TBP, the DNA_unbound_ peak (centered at *P* ≈ 0.15) transitions to a new peak at *P* ≈ 0.5 (DNA_bound_); this can be seen most clearly at 10 nM TBP. At concentrations greater than 10 nM, however, it can be seen that DNA_bound_ starts to spread across multiple proximity ratios, coming to near unity at saturation; in line with the conformational change observed in the bulk data (Figure 1C). In order to analyze this phenomenon, we plotted the distribution width obtained when determining the average peak position (Figure 2E). Therefore, in a two-state system, the distribution width should increase until the *K_d_* is reached (50% bound), at which point it should decrease, and eventually return to a width similar to that of unbound DNA, as the entire population shifts to a single bound state. This is highlighted in Figure 2E, where the distribution width of TATA-14E increases as TBP is added, but then decreases again as the whole population is saturated with TBP; the unbound and bound states have nearly identical distribution widths. Conversely, the distribution width for TATA-18I continues to rise throughout the addition of TBP, failing to coalesce into a single narrowly distributed bound state. A broadening of the distribution width indicates that heterogeneity exists in the system, giving rise to multiple proximity ratios. This heterogeneity could arise from each molecule having a discreet proximity ratio or from molecules changing between structural states as they transit through the focus. Considering that this effect is roughly linear with TBP concentration, argues for many states being present in the solution, likely from non-specific binding. These observations are consistent with those from bulk-FRET in Figure 1C.

Considering that increasing the KCl concentration to physiological levels (150 mM) suppressed the biphasic TBP binding to TATA-18I in bulk, we asked whether spFRET could be used to further refine how ionic strength affects TBP binding. Using the TATA-18I DNA construct, we tested TBP binding at 150 mM KCl, with histograms shown in Figure 2C. From these data, we again see two states DNA_unbound_ (*P* ≈ 0.15) and DNA_bound_ (*P* ≈ 0.6) species. Interestingly, upon addition of saturating amounts of TBP, the peak for DNA_bound_ does not broaden nearly as dramatically as in 50 mM KCl. This is highlighted in Figure 2E, where the distribution width increases initially, but then decreases again, signifying a reduced number of conformational states at saturation, compared to 50 mM KCl. It is notable that the distribution width in the DNA_bound_ state remains higher than in the DNA_unbound_ state, indicating it has likely not resolved all of the TBP induced broadening. Regardless, this increase in KCl substantially reduces the broadening observed at 50 mM KCl. Further, calculating average proximity ratios from these data show a normal binding isotherm, as was observed for bulk measurements under these conditions.

As a control for verifying whether double-stranded DNA is required to obtain the concentration-dependent effects responsible for distribution broadening, we performed binding with 200 nM TBP on a construct containing 14 bp of an extra single-stranded DNA extension in replace of the 11 bp double-stranded flanking DNA sequence (Supplementary Figure S4). From these data we do not observe any distinct broadening, showing that double-stranded DNA is required for this effect. Finally, as further validation to test the whether off-consensus binding is responsible for the conformational change; we replicated the binding studies from Figure 1 in the presence of competitor DNA that lacks a TATA box. In this experiment, the competitor DNA was co-titrated with TBP in equimolar concentrations (Supplementary Figure S5). This methodology gives TBP significant spatial opportunity to associate non-specifically with DNA, separate from that containing the TATA box, but not in vast excess as to outcompete specific binding to the TATA-box. Using this approach, we find the biphasic nature of the TBP titration on TATA-14I, at 0 and 50 mM KCl, is eliminated and a single binding isotherm remains. Conversely, the monophasic binding observed at 150 mM KCl remains unchanged upon addition of competitor. This shows that non-specific, off-TATA box, binding is responsible for the reduction in proximity ratio and distribution broadening at reduced ionic strength, while increasing ionic strength suppresses these alternative conformations, facilitating specific TBP recruitment to the TATA box.

### TFIIA enhances TBP specificity toward the TATA box

**Figure 3.**
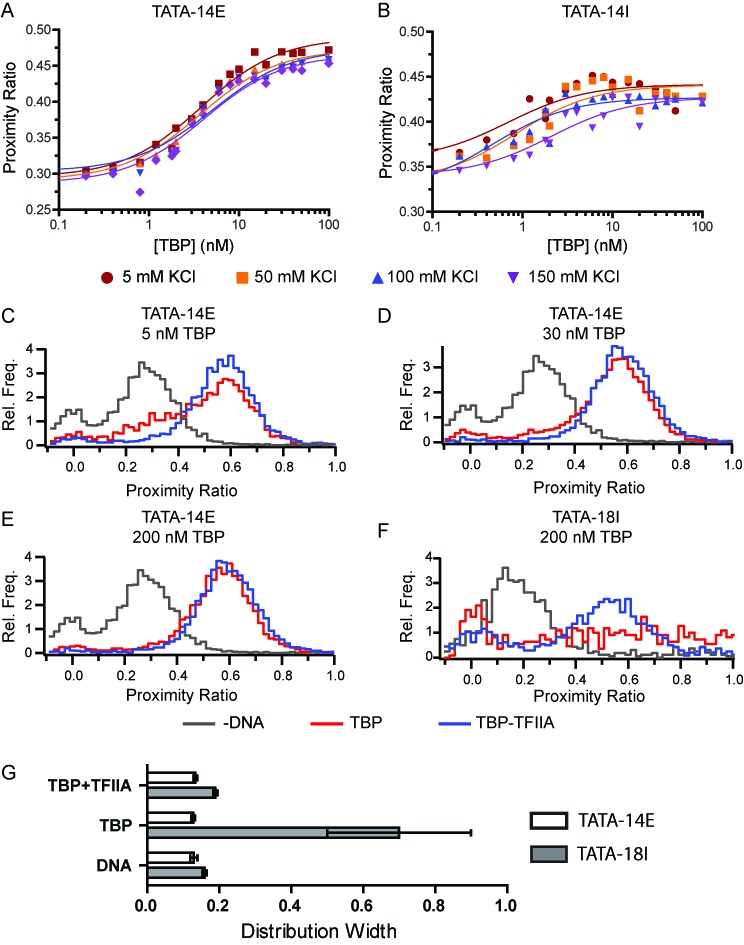
Addition of TFIIA positions TBP over the TATA box. (**A**–**B**) Bulk analysis of TBP-TFIIA binding to (A) TATA-14E and (B) TATA-14I under varying salt concentrations. Data are fit to Equation (1). (**C–F**) proximity ratio histograms obtained using spFRET, comparing binding TBP and TBP with 2-fold molar excess of TFIIA. (**G**) Quantification of distribution widths determined for the histograms shown in for TATA-14E (open bars) and TATA-18I (filled bars) when bound by 200 nM TBP or TBP/TFIIA in 50 mM KCl.

TBP binding stability is salt-dependent (40,46), suggesting that TBP's association to DNA *in vivo* may be quite unstable, even on TATA box DNA. To overcome this instability, the cell uses additional factors, such as TFIIA or NcoI, to stabilize the interaction. Previous studies have shown that TFIIA can dramatically affect the binding properties of TBP to DNA. For example, TFIIA can directionally orient TBP about the TATA box (17), increase specificity to certain sequences (9,47), as well as stabilize the binding of TBP to DNA (10,17). Therefore, we asked whether TFIIA could also influence the non-specific binding behavior observed for TBP on longer DNA constructs. To test whether TFIIA could help modify the biphasic binding behavior, TBP and TFIIA were co-titrated onto TATA-14E and TATA-18I constructs in bulk. Figure 3A shows that TATA-14E is bound by TBP-TFIIA very tightly and follows a normal binding isotherm under all salt concentrations. Notably, the change in proximity ratio is less than that for TBP alone, as previously observed (10). Interestingly, the plateau of the titration series for TATA-14E is salt-independent, unlike TBP alone (Figure 1A), which decreases upon salt concentration. Likewise, the binding curves for TATA-14I (Figure 3B) also mimic those with TBP alone (Figure 1B), however, the sharp drop to a new plateau is clearly subdued.

We next applied spFRET on the TATA-14E and TATA-18I DNA constructs to understand how TFIIA has altered the binding conformation of TBP. We initially tested TBP binding to TATA-14E in the absence or presence of TFIIA at 50 mM KCl. At 5 nM TBP-TFIIA (Figure 3C), the DNA appears to be nearly completely bound by TBP-TFIIA, whereas a small peak of unbound DNA remains for TBP alone. This signifies an enhanced binding affinity for the TBP-TFIIA complex compared to TBP alone. At 30 nM, TBP alone shows little remaining unbound DNA (Figure 3D). At 200 nM TBP or TBP-TFIIA, a large excess over their binding affinities, no difference is observed between proximity ratio histograms, showing only a single (bound) species (Figure 3E). In this data we observe no intermediate states or significant alterations in conformation, nor significant non-specific interactions at high protein concentrations. Conversely, when 200 nM TBP was added to TATA-18I, a very broad distribution, nearly uniform across all proximity ratios, is observed (Figure 3F). Interestingly, the addition of TFIIA to this reaction dramatically reduces the histogram width and centers the distribution about one single point, with proximity ratio of 0.55. This is highlighted in Figure 3G, showing a graph of distribution widths comparing TATA-14E (clear) and TATA-18I (gray) under these conditions. This coincides well with the data obtained in bulk, where the biphasic nature of TBP binding TATA-14I is reduced in the presence of TFIIA. This suggests that TFIIA helps to suppress non-specific TBP binding conformations and stabilize one single species about the TATA box.

### Kinetic intermediates are formed during the formation of the TBP-TATA complex

**Figure 4.**
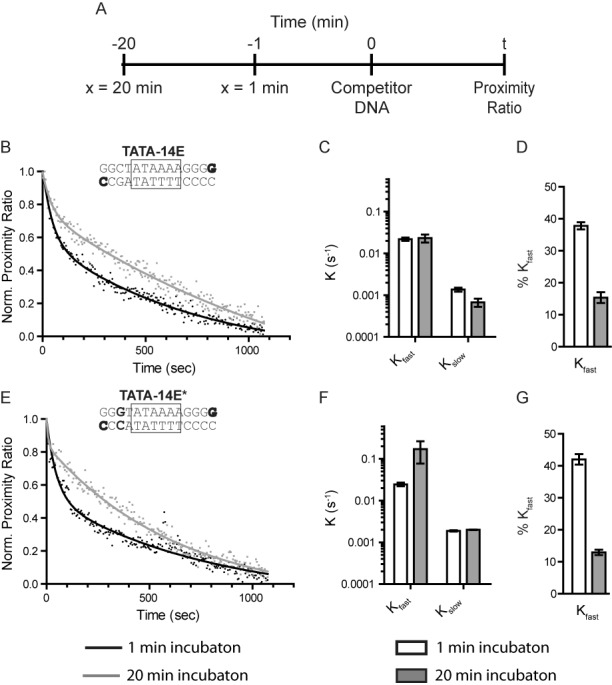
Binding of TBP to TATA-14E is characterized by two-phase dissociation. (**A**) The reaction scheme used to determine TBP kinetic dissociation rates at various incubation times. Experiments were performed in binding buffer with 50 mM KCl. (**B**) Kinetic dissociation curves for TATA-14E when incubated with TBP for 1 (black) or 20 min (gray) prior to competitor addition. Each data set is fit to a two-phase exponential decay. (**C**) A plot showing the dissociation rates obtained from fitting the data in (B) at 1 min (open bars) or 20 min (filled bars). (**D**) A plot showing the fraction of fast component obtained from fitting the data in (B) for 1 min (open bars) or 20 min (filled bars) incubation periods. (**E–G**) Same as in B–D, except using TATA-14E*. Error bars represent the range of values between two independent experiments.

Previous data have postulated that kinetic intermediates form during the process of yeast TBP binding (16,38,48), while other data with human TBP show simultaneous binding and bending of the DNA (36) but with a pronounced biphasic dissociation. Since we did not observe any intermediates on TATA-14E using spFRET, it is highly suggestive that a structural intermediate during binding is not formed, as it would be most highly pronounced at the most dilute TBP concentrations. This implies that TBP may bind in a branched pathway, to which only one species is observed at equilibrium. To test whether multiple distinct species exist, we measured dissociation kinetics after various incubation times to see if we could identify important intermediate binding states. We followed the reaction scheme in Figure 4A, where TBP was incubated with TATA-14E DNA for 1 or 20 min, followed by the addition of excess unlabeled TATA-14 DNA as a competitor. Kinetic dissociation was then observed as a loss in proximity ratio. Using this methodology, we find that after 1 min of incubation, two kinetically distinct dissociation rates are obtained (Figure 4B and C; *K*_slow_ = 0.0014 s^−1^; *K*_fast_ = 0.02 s^−1^), with the fast rate representing a significant portion of the population. After 20 min of incubation, only a small portion of the fast rate remains (Figure 4D, 15% compared to 38%), with the fast and slow rate constants (*K*_slow_ = 0.007 s^−1^; *K*_fast_ = 0.02 s^−1^) virtually identical to those after 1 min incubation. The dominance of a biphasic curve for 1 min incubation is evident when plotting the residual of single- and two-phase exponential curve fits (Supplementary Figure S6). In contrast, the residuals at the 20-min point appear quite similar, other than the first few time points, suggesting little if any fast phase. As a control, this experiment was also performed on TATA-14E*, showing nearly identical results (Figure 4E–G). This shows that a minor change of G to C does not affect these rates or proportions. The observation of two binding constants with distinctly different rates is strikingly similar to those obtained recently on single immobilized DNA molecules (0.3 s^−1^) (37), while the slow rate constant is nearly identical to that previously obtained for the consensus TATA box in bulk (0.002 s^−1^) (10). Notably, the fast rate constant is reminiscent of dissociation rates obtained for a mutated TATA box (*k*_off_ = 0.06 s^−1^) (10). Together, these data show that TBP initially binds in either two or more distinct conformations or locations on the DNA, but eventually reaches equilibrium at the kinetically stable TATA box.

### TBP accessibility to the nucleosome is salt-dependent

**Figure 5.**
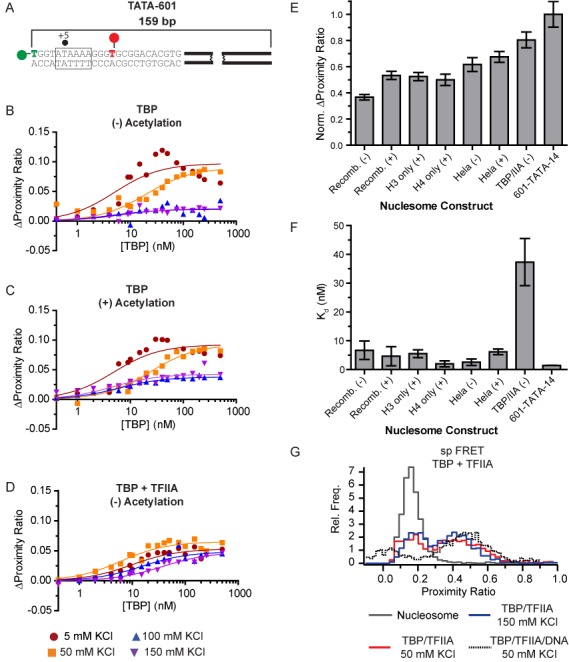
TBP binding is severely restricted at the DNA entry–exit sites of the nucleosome. (**A**) Schematic showing the TATA-601 DNA construct used for nucleosome binding studies. (**B** and **C**) Plots showing a change in proximity ratio to TATA-601 nucleosomes, with increasing TBP concentration and varying KCl. Nucleosomes were either (B) non-acetylated (−), (C) chemically acetylated (+) or (D) in the presence of 2-fold molar excess of TFIIA over TBP. **(**E**)** A bar plot showing changes in proximity ratio for TBP binding to non-acetylated (−) or acetylated (+) nucleosome constructs. Data were normalized to the change in proximity ratio when binding naked TATA-601 DNA at 150 mM KCl. For nucleosomes, total change in proximity ratio includes the additional bend incurred by DNA wrapping on the nucleosome. Full binding curves can be seen in Supplementary Figure S8. (**F**) A bar plot showing the relative affinities measured for TBP binding to the indicated nucleosome constructs in 150 mM KCl. (**G**) spFRET histograms comparing TBP and TBP-TFIIA binding to naked or nucleosomal TATA-601 DNA. TBP and TFIIA were added at 200 nM each for naked DNA, while 100 nM TBP and 200 nM TFIIA were used for nucleosomal DNA.

In the cell, TBP is not generally exposed to naked DNA, but must overcome a barrier imposed by nucleosomes. Nucleosomes are used to suppress non-specific, as well as specific, TBP association. This is highlighted by studies showing PIC formation at TATA box promoters to be competitive with nucleosome occupancy, requiring displacement of the nucleosome to attain transcription activation (21). Data suggest that the nucleosome may be judiciously placed with respect to the TATA box and that the dynamic nature of the nucleosome entry–exit site may be used to modulate TBP's (or other transcription factor's) access to the promoter. This is consistent with previous studies showing both position- and phasing-dependent suppression of TBP binding to the TATA box within a nucleosome (26). This leads to questions how the nucleosome is correlated with both recruitment and repression of TBP binding to its intended target site. Therefore, we utilized the experimental methodologies detailed in the previous sections to measure, in real-time, TBP accessibility to nucleosome occupied DNA. To do so, we designed the TATA-601 DNA construct to directly measure TBP accessibility in solution, where TATA-601 is a 159 bp DNA based upon the Widom ‘601’ positioning sequence (Figure 5A and Supplementary Figure S7) (49). In this sequence, a TATA box was centered ∼5 bp inside the positioning sequence, such that the minor groove associated with the H3 N-terminal helix would compete for TBP binding. Due to the natural fluctuations in this region, this position is highly dynamic and represents the most accessible site within the context of the nucleosome, and would give the greatest opportunity for TBP to bind competitively (28,50,51).

In order to understand the salt-dependent competition between nucleosomes and TBP, this construct was tested for TBP's ability to bind the inserted TATA box at varying salt concentrations in the same manner as the naked DNA constructs in Figure 1. As seen in Figure 5B and Supplementary Figure S8, nucleosomes show a binding pattern nearly identical to naked DNA at 5 and 50 mM KCl, suggesting both specific and non-specific binding is easily established under low ionic strength. However, binding studied at 100 and 150 mM KCl are severely reduced in maximal proximity ratio upon saturation, relative to naked DNA (Figure 5E). This shows the nucleosome is a potent inhibitor of TATA box specific TBP binding under physiological ionic strength. Of note, the signal change at 150 mM KCl is not marked by a prominent increase in *K_d_* (Figure 5F), indicating the small amount of signal present may be due to off consensus binding or heterogeneity in the nucleosomes; notably, our nucleosome preparations appear homogeneous with relatively little free DNA, as viewed by native polyacrylamide gel electrophoresis (Supplementary Figure S9). This partial accessibility is consistent with DNA digestion studies of the nucleosome (50) and may suggest that the 601 positioning sequence adopts more than one unique conformational state. Regardless, this shows that the nucleosome poses as a steric block to complete TBP binding to the TATA box, even at the most exposed site.

### Salt-dependent architectural changes in the nucleosome regulate competitive association with the TBP-TFIIA complex

In bulk, the addition of TFIIA with TBP shows a consistent pattern of enhanced affinity and specificity toward the TATA box on naked DNA. This suggests that TFIIA may play a crucial role in facilitating specific recruitment of TBP to the TATA box within the nucleosome entry–exit site. To further elucidate whether TFIIA plays a functional role in specific recruitment of TBP to nucleosome occupied DNA, under physiological conditions, we tested whether TFIIA enhances the ability of TBP to bind and bend the TATA box sequence within a TATA-601 nucleosome. In Figure 5D and Supplementary Figure S8, TFIIA clearly enhanced binding relative to TBP alone at 150 mM KCl, with proximity ratios nearly equivalent to those on naked DNA (Figure 5E). Unlike TBP alone, we also found a salt-dependent shift in apparent binding affinity (Figure 5F and Supplementary Figure S10), displaying competitive association with the nucleosome, as one would for a sequence-specific binding protein. To further probe whether the conformation of the TBP-TFIIA complex to TATA-601 is altered within the nucleosome, we used spFRET to compare architectures in absence and presence of the nucleosome (Figure 5G). From these data, we observe no significant deviation in the presence of the nucleosome compared to naked DNA. This shows the complex is binding in the fully bound and bent state.

Considering the large bend induced on the DNA by TBP binding, we questioned whether the TBP-TFIIA complex can promote global architectural changes within the nucleosome, which could be used to enhance the ability of additional factors to bind. To do so, we used spFRET to observe changes in the histone DNA contacts upon TBP-TFIIA binding to the TATA box within TATA-601 nucleosome, as described previously (33). From these spFRET traces (Supplementary Figure S11 and Supplementary Results), we observe only a slight change in the nucleosome structure upon TBP-TFIIA binding at 5 mM but not at 150 mM KCl. This is a similar difference observed by increasing salt between 5 and 150 mM KCl, consistent with other observations showing salt-dependent architectural changes in the nucleosome (32,52,53). These data suggest the entry–exit sight is highly flexible under both ionic strengths and its architecture has little impact on the global structure of the nucleosome.

### Histone acetylation enhances TBP accessibility to nucleosomes

Post-translational modifications (PTMs) of histones are highly correlated with a variety of activities within the cell, with histone acetylation being directly linked to increased transcriptional activity (30). Various data show that acetylation changes nucleosome accessibility by neutralizing charges associated with DNA binding, making it more permissible to transcription factor binding. To test the role of acetylation on nucleosome architecture and TBP accessibility, we constructed nucleosomes on TATA-601 that contained chemically acetylated histones on all four histones, only histone H3, or only histone H4. When binding TBP to hyper-acetylated nucleosomes, at low ionic strength, we obtained binding curves comparable to those obtained under non-acetylated conditions (Figure 5C), suggesting high accessibility to specific and non-specific binding events. However, at 150 mM KCl, accessibility is significantly enhanced by the presence of bulk acetylation (Figure 5F). Notably, binding affinity remains high for these curves, suggesting a fraction of the nucleosomes contain acetylation states amenable to increased binding while a fraction remains reflective of non-acetylated nucleosomes. Further refinement shows that acetylation of only histone H3 or only histone H4 results in nearly equivalent accessibility as bulk acetylation of all histones. This indicates that histones H3 and H4 are primarily responsible for regulating nucleosome accessibility at the entry–exit site.

Historically, endogenous histones have been used to perform many studies measuring nucleosome accessibility; however, more recent studies have more frequently relied on recombinant histones. Given endogenous histones may harbor modifications other than histone acetylation and that they may be placed more judiciously on the histones, we questioned whether endogenous histones form more permissive nucleosomes. To test this possibility, we constructed nucleosomes containing histone octamers isolated from HeLa cells that contained either ‘normal’ modification patterns or were hyper-acetylated by incubating cells with sodium butyrate. These nucleosomes were then tested for the ability of TBP to bind the TATA box within TATA-601 nucleosomes (Figure 5E and Supplementary Figure S8). Consistent with our previous results, TBP can easily bind the entry–exit site of the nucleosome under reduced ionic strength. Strikingly, at 150 mM KCl, HeLa derived nucleosomes retain the highest maximal proximity ratio compared to any recombinant nucleosomes, while hyper-acetylation of these nucleosomes produces only a modest additional increase. Similar to recombinant histones, binding affinity remains unchanged (Figure 5F). These data suggest native PTM patterns are judiciously placed to have a stronger influence over nucleosome architecture than acetylation alone.

Overall, our data show that ionic strength plays a large role on nucleosome architecture and accessibility to TBP. This is highlighted by physiological ionic strength suppression of non-specific TBP association to naked DNA, while specific TATA box association is blocked by the nucleosome. Considering that the nucleosome is permissive under low ionic strength, this suggests external factors or PTMs are able to play a similar role as the salt by promoting enhanced nucleosome accessibility.

## DISCUSSION

TBP binding to DNA has been thoroughly characterized in bulk (8,16,17,36,54), but many questions remain about what differentiates TBP binding between TATA-containing and TATA-less promoters, and how the nucleosome barrier is overcome. Here we combined bulk and single-molecule FRET experiments to characterize TBP-DNA association in real time; to date few studies have looked at TBP binding in detail using single-molecule imaging (37,55). We determined that short DNA sequences containing a TATA box are bound by TBP with high specificity and binding appears as two states (unbound and bound) when observed under single-molecule conditions. However, the addition of flanking DNA sequence, adjacent to the TATA box, fundamentally alters the DNA conformational landscape, increasing the heterogeneity of bound states, indicative of non-specific binding. Reduction in non-specific binding can be achieved by increasing the salt concentration to physiological levels and/or the addition of TFIIA. This single-molecule data, combined with kinetic experiments, show that TBP binds via a branched pathway, where TBP initially associates with little sequence specificity, but re-equilibrates to the stable TATA box over time. The incorporation of a TATA box near the entry–exit site of a nucleosome suppresses TATA box specific TBP binding under physiological conditions, where acetylation of histones H3 or H4, or addition of TFIIA, alleviates some, but not all, repression. Together our data show that TBP, *in vivo*, would rarely be engaged long enough on the DNA to facilitate cryptic transcription, but requires additional protein factors and changes to the nucleosome architecture to direct sequence-specific localization.

### TBP binds the TATA-box as a single uniform species

Our single-molecule FRET results clearly show that TBP binds to the consensus TATA box in a two-state function. This meaning, within the resolution of our measurements, we observe no intermediate FRET states between the unbound and fully bound/bent states on a minimal 14 bp DNA. These experiments were performed under a variety of TBP concentrations, where, if any intermediate state were to be present during formation of the fully bound/bent state, it would be most prominent at subsaturating (dilute) TBP concentrations. Under subsaturating conditions, we find no evidence for additional states. This result is consistent with recent spFRET results by Blair *et al.* showing only unbent and fully bent species using single-molecule TIRF imaging (37). Our data are also consistent with a previous model by Parkhurst *et al.*, using fluorescence lifetime measurements with a similar FRET system, suggesting simultaneous binding and bending by human TBP to TATA box DNA (12).

### Flanking DNA fundamentally alters the TBP-dependent DNA conformation

Previous data have shown that DNA flanking the TATA box plays an important role in defining TBP sequence-specific thermodynamic and kinetic binding parameters. To approach this question, we added 11 bp of flanking DNA next to a minimal 14 bp TATA box DNA, and probed the thermodynamic and conformational effects induced by this additional sequence. From this, we found that TBP binds specifically to the TATA box under dilute TBP concentrations, at equilibrium. However, as the TBP concentration was increased, we observed a fundamental change in the architecture of the DNA in both bulk and spFRET measurements. Within our spFRET measurements, this change was characterized by a roughly linear increase in proximity ratio distribution with TBP concentration, while bulk values show a rapid reduction in proximity ratio. Due to the concentration-dependent nature of effects, we attribute these phenomena to non-specific binding of TBP to this DNA construct. This is further validated by our observation that non-specific association is dramatically suppressed by increasing the monovalent salt concentration to 150 mM, suggesting that *in vivo*, TBP is rarely bound for any significant lifetime at non-consensus sites; a similar affect is observed by adding competitor DNA to suppress non-specific interactions.

These data are consistent with footprinting studies by Coleman and Pugh showing TBP is able to saturate a long DNA template, with non-consensus sites more prone to digestion (56). Additionally, Kays and Shepartz found diffuse patterns for TBP association about the TATA box using a DNA cleavage agent attached to TBP (57). Interestingly, the transition between specific and non-specific association occurs at relatively low TBP concentrations, near 10 nM. Considering the binding affinity of TBP to the consensus TATA box is ∼2 nM at 50 mM KCl, this highlights that there is not strong specificity toward the TATA box under low Ionic strength. Due to the large number of potential binding sites, even on a short DNA fragment, the level of specificity is significantly less than that obtained from previous studies showing 10- to 100-fold differences in affinity and kinetic stability between consensus and mutated TATA sequences, suggesting other energetic factors are at play (10,58). Given this low level of specificity, these data explain how mutations introduced into the TATA box could lead to an increase in the distribution of FRET states (13). For example, if a mutation is introduced within the TATA box, the affinity and specificity for the intended sequence is reduced, thus increasing the likelihood of off-target binding. Off-target binding would lead to different proximity ratios for each site bound, resulting in a broad distribution of values with the potential for changes in the average measured proximity ratio.

### TBP's inability to recognize specific sequences upon association defines a branched pathway to TATA-box recognition.

Using kinetic-based bulk FRET measurements, we find that TBP dissociates from a minimal TATA-box fragment with a biphasic signature. Upon testing dissociation after short and long incubation periods, we find the mole fraction of the fast phase decreases over time. Interestingly, the rates for fast and slow species remain constant regardless of incubation time. Our data are consistent with previous observations that human TBP contains multiphasic dissociation kinetics (12,37). Delgadillo *et al.* applied global analysis to their thermodynamic and kinetic measurements, arriving at a three-step linear binding mechanism, defining the transition from unbound to a fully bound and bent TBP-DNA complex (16). This pathway is initially characterized by two intermediates that are structurally indiscernible from the final state but show kinetic representation. In contrast, recent spFRET work by Blair *et al.*, has observed the kinetic formation of TBP to DNA, in real-time, and found only two distinct kinetic dissociation rates and a single exponential association rate (37). Using these data they developed a revised model for TBP binding, wherein their data reflect a mechanism with two distinct states that are structurally indistinguishable from the final bound/bent state. They postulate these states could arise from a linear or branched pathway, as defined in Reaction Scheme 1, but from their data could not differentiate between each.

**Figure 6.**
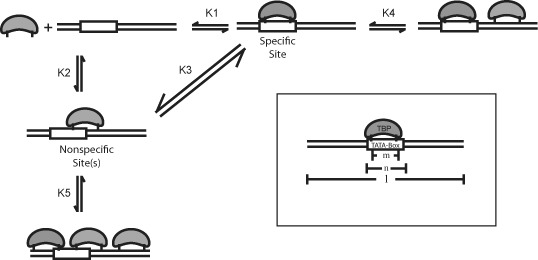
Non-specific binding of TBP to DNA gives a branched-pathway to binding. Branched binding mechanism deduced for TBP binding to DNA. K1 is the affinity to the TATA box. K2 is affinity to all other possible binding sites. K3 represents TBP sliding from site-to-site without dissociation sliding between sites, as described by Pugh *et al.* (8). K4 represents the addition of a second TBP binding to a DNA with an occupied TATA box. K5 represents the addition of a second TBP binding to DNA which is bound by TBP off the TATA box. Inset: A schematic showing the factors limiting the number of positions and number of molecules that can bind a limited DNA lattice. ($\ell$$\ell$) is the base length of the DNA, (*m*) is the furthest DNA length between protein-DNA contacts and (*n*) is the length of DNA length covered by the protein's mass.

By incorporating the new results presented here, showing that TBP binds a variety of sequences and that biphasic dissociation is time-dependent, with the previous results we are able to further refine the TBP binding model. From these data, we conclude that TBP binding follows a branched pathway (Figure 6), wherein TBP initially binds to many (*K*_2_) or all, possible DNA sequences with roughly equal forward association rates (total of 14 sites), since only one TBP molecule may bind at a time. After this initial association event, TBP then slowly transitions to the most kinetically stable site (*K*_1_), the TATA box, by going through multiple rounds of association and dissociation. This would be consistent with Blair's observation that TBP association proceeds with a single uniform association rate despite its biphasic dissociation profile. Given the short DNA length, only small differences in DNA end-to-end length would be expected from shifting TBP binding by 2–3 bp, making it difficult to differentiate between species, which is consistent with the previous bulk and spFRET studies. Our model suggests TBP shows little discrimination between sequences during association and sequence specificity is defined by the sequence's kinetic stability. This agrees with the observation that the first phase of TBP binding (in the three-step model) is independent of TBP concentration, as would be expected in our model (42).

While our data clarify the use of branched pathway for TBP binding, we also cannot rule out that an additional linear pathway is followed, characterized by initial formation of an unbent (transient encounter) intermediate, as previously suggested (12). The presence of an unbent intermediate may be quite likely, considering a 10- to 100-fold increase in TBP association rate was observed using FRET measurements between TBP and the DNA relative to those measuring bending (59). These rate constants are more consistent with diffusion limited association rates compared to the slow association rate of 2 × 10^6^ M^−1^s^−1^ for TBP bending at the consensus site. Non-bending association would also be consistent with the observation that TBP can slide on the DNA (*K*_3_), where insertion of two phenylalanine residues between bases on each side of a 6 bp stretch of DNA would not be amenable to sliding, but unbent ‘loose’ association would be. Further studies measuring association between TBP and the DNA will be needed to address this issue.

### Cooperative non-specific TBP association out-competes specific TATA box recognition

As detailed above, the addition of DNA adjacent to the TATA box results in competition between non-specific TBP association and specific association to the TATA box, in a concentration-dependent manner. In theory, the fraction of non-specific and specific interactions should be proportional to their binding affinities. However, we find this is not the case under elevated TBP concentrations. Therefore, we further refined the kinetically defined branched pathway detailed above, proposing that the ability to compete for specific TATA box binding is a result of cooperative TBP association (Figure 6); based upon the McGhee/von Hippel model for non-specific binding (60). In this model, TBP initially binds TATA box DNA with roughly equal forward association rates to many, or all, possible DNA sequences, including the consensus TATA box (*K*_2_). Thereafter, TBP slowly transitions to the most energetically stable site(s) (*K*_1_). At dilute TBP concentrations binding would be directed toward the TATA box. However, under elevated TBP concentrations, more TBP molecules are able to associate on the long DNA polymer (*K*_5_), resulting in a net energetic favorability for multiple binding events relative to binding TBP the TATA box (*K*_4_). This is highlighted in our data by specific binding at dilute TBP concentrations, but with a dramatic decrease in proximity ratio and distribution broadening as multiple TBP molecules associate on the DNA. To explain this effect, this suggests that a cooperative effect between TBP molecules to non-specific sites, where once one TBP molecule binds to an off-consensus site it increases the likelihood of a second molecule binding to another off-site sequence. This could occur simply through the energetic favorability of two molecules being bound, via intermolecular interactions between TBP molecules, or where bending induced from one TBP molecule may influence the binding of a second. Regardless, this model can help to explain how neighboring sequences are able to differentially influence TBP's binding affinity and stability (54). For example, neighboring sequences may result in a more or less stable TBP interaction near the TATA box, which in turn may result in phasing of TBP molecules either on or off the consensus TATA box. Thus, dependent on this phasing, kinetic and thermodynamic stability profiles would be affected differentially at low and high TBP concentrations.

The model in Figure 6 can help explain previous observations regarding TBP binding and specificity. For example, our data show that adding TFIIA to the system significantly suppresses non-specific binding. This suggests that TFIIA adds DNA sequence selectivity to TATA box binding and/or suppresses cooperative association between TBP molecules, enhancing affinity toward the specific site and reducing the impact of non-specific saturation on the DNA. This is consistent with *in vivo* results which show large effects in transcription upon minor changes in TATA box sequence (47), as well as *in vitro* results, showing that TBP binding to the TATA box is stabilized by TFIIA (10,17). Kays and Schepartz have shown similar results to ours, observing diffuse TBP binding patterns about the TATA box that are narrowed in presence of TFIIA (57). Furthermore, Lamb *et al.* have tested TBP binding under single-molecule conditions, and observed a variety of proximity ratios arise between labeled TBP and labeled DNA (55). These distinct states appear to have a periodicity to them, wherein the highest probably is centered about the TATA box, and the other peaks taper off in a Gaussian fashion. This variety of states likely arises from off-TATA box binding by TBP, because in their experiments, addition of TFIIA strongly reduces these alternative states, similar to our data.

### Salt-dependent nucleosome architecture regulates TBP accessibility

In a cellular environment, TBP must not only recognize its intended binding site sequence-specifically, but also overcome the nucleosome barrier. Previous studies have shown that nucleosome remodeling is required for TBP to gain access to nucleosome occupied promoter DNA; however, it is unclear whether the nucleosome must be completely removed from TBP's binding site, or whether nucleation may begin when it is partially buried within the nucleosome. Given previous observations that the DNA entry–exit site of the nucleosome is highly dynamic, suggests that the final histone contacts within the nucleosome may act as a key regulator to transcription factor binding, including that of TBP. Using FRET experiments with a TATA box buried 5 bp inside the nucleosome, at the location where the minor groove containing the last histone contacts, we observed strong binding to both specific and non-specific sites under low ionic strength; reminiscent of naked DNA binding. In contrast, elevated ionic strength (150 mM KCl) resulted in a strong reduction in TBP binding, driving it to bind off-consensus outside of the nucleosome contacts. In contrast, the addition of TFIIA increased specific binding to the TATA box, and showed a pattern of reduced affinity suggestive of competition with the nucleosomal interactions. These results are consistent with previous experiments showing strong inhibition of TBP binding in this region, using DNAse I footprinting (26,27). These studies, performed under low ionic strength, also observed broad footprinting patterns with TBP alone, and would be reminiscent of the non-specific coating of the exposed DNA by multiple TBP molecules.

From these data, we would like to point out two interesting observations. First, in theory, TBP should be able to out-compete the nucleosome for DNA occupancy and eventually reach complete binding and bending of the DNA. Our data show that TBP alone is incapable of fully binding and bending the partially buried TATA box DNA within the nucleosome, under physiological ionic strength. The addition of TFIIA, however, does produce competitive behavior. Therefore, TBP binding does not compete with DNA-histone contacts, as observed for LexA binding (29), but rather the nucleosome appears to dictate where TBP can bind. This suggests that the nucleosome has the capacity to restrict bending, dislodge TBP and/or push TBP away from the histone occupied region. While we cannot differentiate between these models, these data add the possibility that the nucleosome can alter the stability patterns of transcription factor binding, acting as a more potent inhibitor to binding than a simple competitor. In contrast, the addition of TFIIA followed a more traditional competitive binding pattern, suggesting the nucleosome cannot displace this strong interaction, once formed. Notably, the nucleosome appears to have the ability to allow TBP association to TATA-less sequences to an area just outside the nucleosome with relatively high affinity. This may explain how TFIID-dependent recruitment of TBP to TATA-less promoters is correlated with specific nucleosome occupancy (21).

The second interesting result we obtained from these experiments is that TBP accessibility to the nucleosome is salt-dependent. Our data show that the entry/exit site changes conformation based upon ionic strength, wherein the DNA behaves as though it is naked at low salt concentration, but under higher, near physiological conditions, it is highly repressive to TATA box binding/bending. Further analysis using spFRET did not reveal significant salt-dependent architectural changes within the core of the nucleosome, suggesting that the entry–exit site is the primary region of modularity. Others have observed salt-dependent conformational changes in the nucleosome in the 50–150 mM KCl (32,61–63), suggesting the nucleosome weakens its contacts within the entry–exit site of the nucleosome under physiological conditions (32–34,52). This gives further evidence that this region of the nucleosome is highly flexible and can adopt multiple conformations without changing the rest of the nucleosome's architecture. Despite the nucleosome loosening its grip at the ends under physiological conditions, these data suggest it may be the dynamic nature of the entry–exit site that is needed for enhanced removal of TBP from the TATA box. Clearly, further studies will be needed to determine whether nucleosome dynamics facilitate transcription factor removal.

### Histone PTM's facilitate TBP binding within the nucleosome entry–exit site

Given our observation that changes in the ionic environment around the nucleosome can alter the conformation of the nucleosome entry–exit site, we questioned whether inherent changes in the nucleosome composition can achieve the same effect. Histone PTMs are a key means by which nucleosome architecture can be altered and may be able to affect transcription factor binding in the nucleosome entry–exit site. One key modification associated with enhanced histone turnover and increased transcriptional activity is the histone acetylation (19) which is also coupled to SAGA-dependent remodeling of the nucleosome (64). By measuring TBP binding to bulk acetylated histones, we find TBP gains enhanced access to the nucleosome under physiological ionic strength. Further analysis of using individually modified histones H3 and H4 show comparable increases in accessibility to bulk acetylation. This suggests that histones H3 and H4 are primarily responsible for granting access to the nucleosome in the entry–exit site. These data are consistent with previous footprinting data showing that histone acetylation enhances TBP accessibility to nucleosomal DNA (27). Interestingly, we do not gain the same change in proximity ratio as on naked DNA, suggesting the nucleosome still retains the capacity to limit binding and bending of the TATA box. We interpret this as that acetylation may simply increase the depth or degree of bend by which TBP can bind within the entry–exit site or that a fraction of nucleosomes have enhanced propensity to invasion.

In addition to testing the role of acetylation on recombinant histones, we also questioned whether endogenous histones from HeLa cells behave differently. By characterizing TBP binding to nucleosomes containing endogenous HeLa histones, we find enhanced accessibility beyond chemical acetylation alone; further chemical acetylation leads to no additional enhancement. This shows that endogenous histones, likely containing modifications that are more potent regulators of nucleosome accessibility than acetylation alone. This lends credence that modifications judiciously placed on the histones can regulate accessibility. Thus, complete removal of the nucleosome is not necessary for TBP to access its cognate site, but a combination of factors, such as phasing of the TATA box within the nucleosome by remodeling factors, incorporations of histone modifications and addition of TBP stabilizing factors, such as TFIIA, can help recruit TBP to its recognition site on the promoter.

Our observations have shed new light on TBP binding and its role in the regulation of transcription. As many different components are continually fighting for limited space on genomic DNA, the cell has accommodated multiple mechanisms to direct binding of specific proteins to distinct sites within the genome. Our data imply that TBP, in the cell, binds rather unstably to a variety of sequences and TBP alone does little to define the PIC location by itself, but works with other factors that modulate chromatin structure and utilize TBP's unique binding properties to direct it to the site of interest. By doing so, the cell suppresses cryptic initiation, while being able to specify the direct location for gene-specific transcription. It is apparent that specific TBP binding is a rather complex event, and further high-resolution studies will be necessary to elucidate the mechanistic details into specific TBP association at promoters.

## SUPPLEMENTARY DATA

Supplementary Data are available at NAR Online.

SUPPLEMENTARY DATA
