# Supplementary material for: The conformational state of the nucleosome entry–exit site modulates TATA box-specific TBP binding
Source: Nucleic Acids Res. 2014 May 14;42(12):7561–76. doi: 10.1093/nar/gku423 (PMC4081063; doi:10.1093/nar/gku423)
Supplement: SUPPLEMENTARY DATA [file supp_42_12_7561__index.html]

The conformational state of the nucleosome entry–exit site modulates TATA box-specific TBP binding — SUPPLEMENTARY DATA 

# The conformational state of the nucleosome entry–exit site modulates TATA box-specific TBP binding

## SUPPLEMENTARY DATA

**Files in this Data Supplement:**

- SUPPLEMENTARY DATA
