# Supplementary material for: The conformational state of the nucleosome entry–exit site modulates TATA box-specific TBP binding
Source: Nucleic Acids Res. 2014 May 14;42(12):7561–76. doi: 10.1093/nar/gku423 (PMC4081063; doi:10.1093/nar/gku423)
Supplement: SUPPLEMENTARY DATA [file supp_gku423_nar-00803-m-2014-File008.pdf]

The conformational state of the nucleosome entry-exit site modulates TATA box specific TBP binding

Authors: Aaron R Hieb[1], Alexander Gansen, Vera Böhm, Jörg Langowski*

SUPPLEMENTARY RESULTS

Nucleosome entry-exit salt dependency – In order to test whether nucleosome conformation changes when bound by TBP, we collected single-molecule traces with FRET pairs between H2B and the DNA at two locations, near the dyad (H2B-15) and adjacent to H2A-H2B (H2B-52), as described previously (63). In these experiments, a change in ionic strength slightly increases the fraction of no FRET species on the DNA-52, but not DNA-15 constructs (Fig. S11). A similar observation is made when adding TBP-TFIIA to nucleosomes at 5 mM KCl, but not at 150 mM KCl. This shows that TBP association has little influence over the nucleosome's global structure.

SUPPLEMENTARY METHODS

Calculation of Proximity ratio–Proximity ratios were calculated by subtracting background, donor bleed-through into the transfer channel, and direct acceptor excitation, as previously described (64); all intensities were first corrected for background. Briefly:

Donor bleed-through is determined from donor (D; 488 nm excitation, 500-540 nm emission) and FRET signals (F; 488 nm excitation, 655-685 nm emission) with a DNA containing only donor fluorophore.

$$\chi_D = \left(\frac{F}{D}\right)_{\text{Donor Only}} \quad \textbf{(1)}$$

Acceptor direct excitation is then determined from acceptor (A; 633 nm excitation, 655-685 nm emission) and FRET signals with a DNA containing only acceptor fluorophore.

$$\chi_A = \left(\frac{F}{A}\right)_{\text{Acceptor Only}} \quad \textbf{(2)}$$

The corrected intensity in the FRET channel (F_{corr}), for DNA carrying both donor and acceptor fluorophores, is calculated using the following equation:

$$F_{corr} = F - (\chi_D \cdot D) - (\chi_A \cdot A) \quad \textbf{(3)}$$

And the plotted P-value is:

$$P = \frac{F_{corr}}{F_{corr} + D} \quad \textbf{(4)}$$

REFERENCES

65. Vasudevan, D., Chua, E.Y. and Davey, C.A. (2010) Crystal structures of nucleosome core particles containing the '601' strong positioning sequence. *J Mol Biol*, **403**, 1-10.

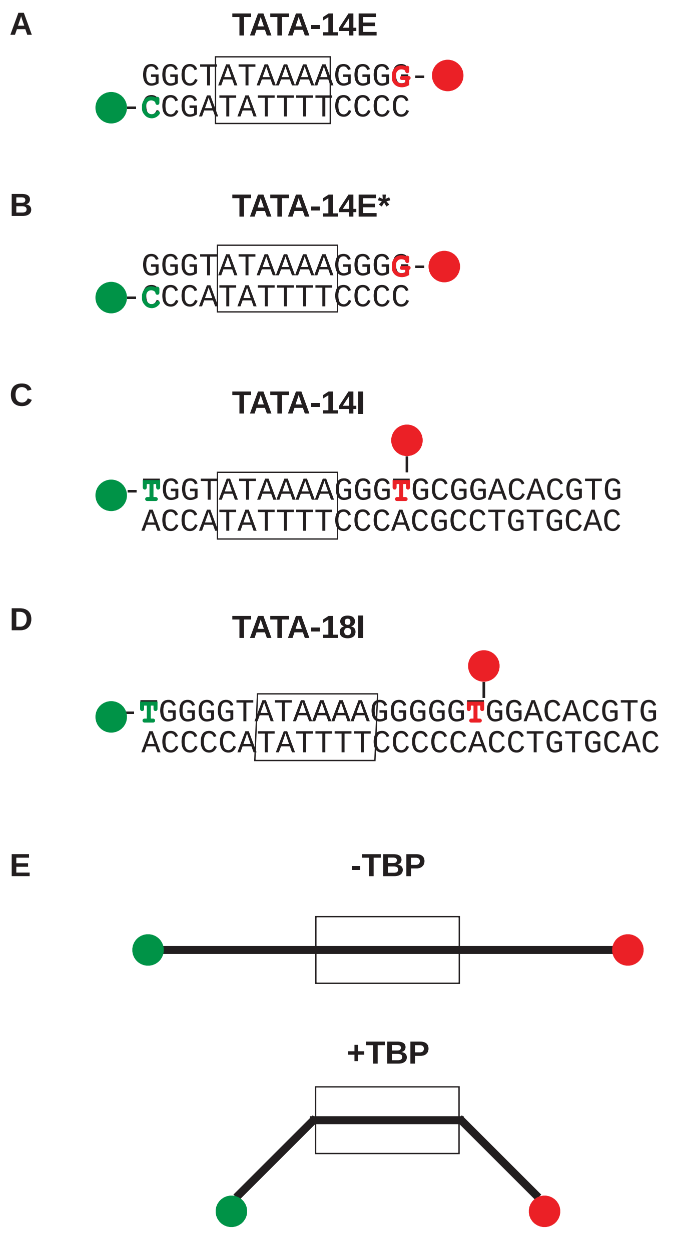

Figure S1. TATA DNA constructs used for TBP binding to DNA. A) and **B)** Short 14 bp constructs for testing TBP binding to DNA. Each is end-labeled with donor (green) and acceptor (red) fluorophores. Upon binding by TBP, the DNA is bent giving rise to an increase in FRET signal. (A) TATA-14E and (B) TATA-14E* differ by one sequence change outside of the TBP binding region. **C)** and **D)** 25 bp DNA constructs which are 5' end-labeled with a donor fluorophore and internally-labeled with an acceptor fluorophore about the TATA-box. TATA-14I (C) and TATA-18I (D) have 14 bp and 18 bp between FRET pairs with total DNA lengths of 25 bp and 27 bp, respectively. **E)** Cartoon representation showing how the FRET pair dyes come closer together upon TBP binding.

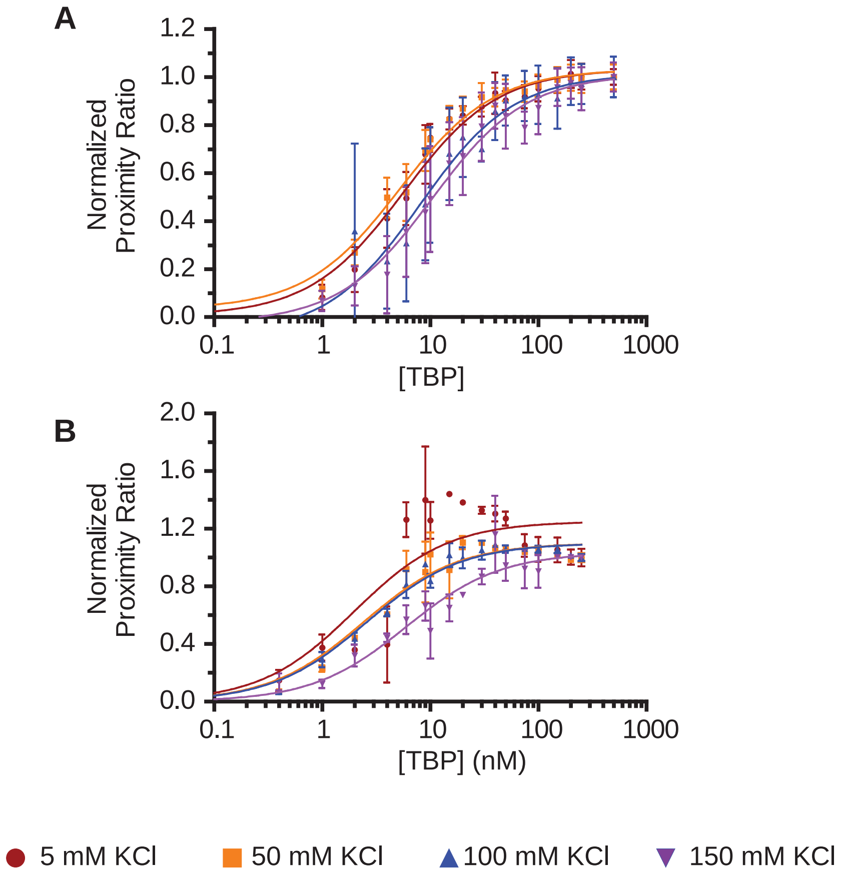

Figure S2. Data from globally plotting and fitting TBP binding to DNA. A-B) show curves for TBP binding to TATA-14E and TATA-14I, respectively. Data points are averages and error bars represent one standard deviation or a range of two values. Notably, some error bars are too small to be seen.

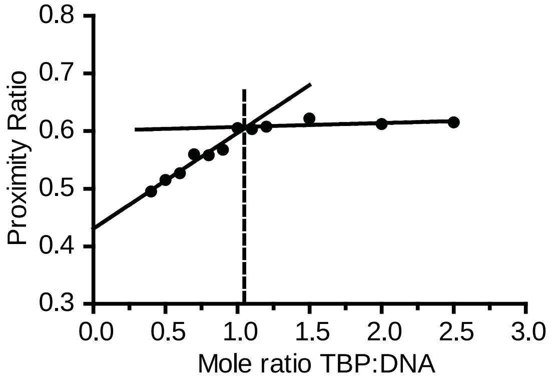

Figure S3. Prepared human TBP binds TATA-14E* stoichiometrically. Shown is a plot of proximity ratio for TATA-14E* upon binding and bending by hTBP, shown as the mole ratio of TBP:DNA. By performing linear regression of each phase of the curve, an intersection point of 1.1 was obtained, signifying 1.1 TBP molecules per DNA fragment. This indicates the TBP is of high spectific activity and only one TBP molecule binds this short DNA fragment.

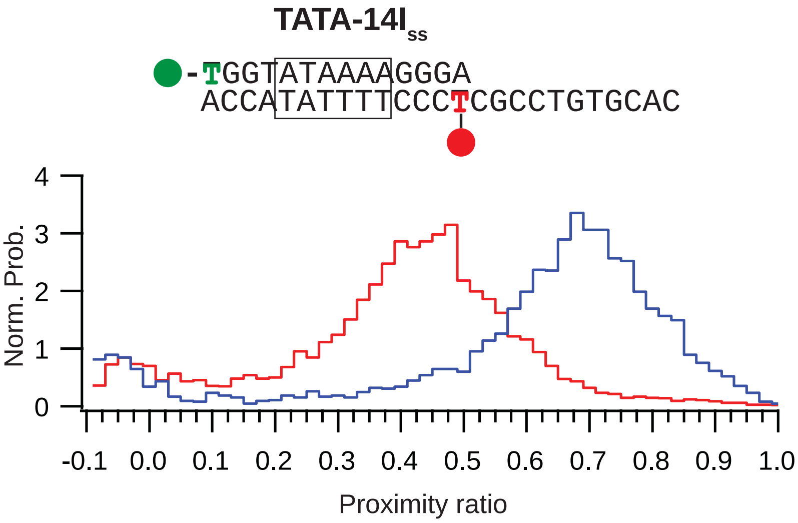

Figure S4. TBP nonspecific binding is dependent upon double-stranded DNA. Upper: Shows a DNA construct (TATA-14I$_{ss}$) used to determine whether flaking double-stranded DNA is required for nonspecific TBP binding. This construct is 25 bp long, but contains single-stranded DNA adjacent to the TATA-box, which allows TBP to diffuse along the DNA but not compete for specific TATA binding. Lower: spFRET histograms showing unbound (red) or TBP bound (blue; 100 nM) TATA-14I$_{ss}$ DNA. We observe no significant broadening of the distribution widths at this high TBP concentration, indicating a dependence on flanking double-stranded DNA toward non-specific binding.

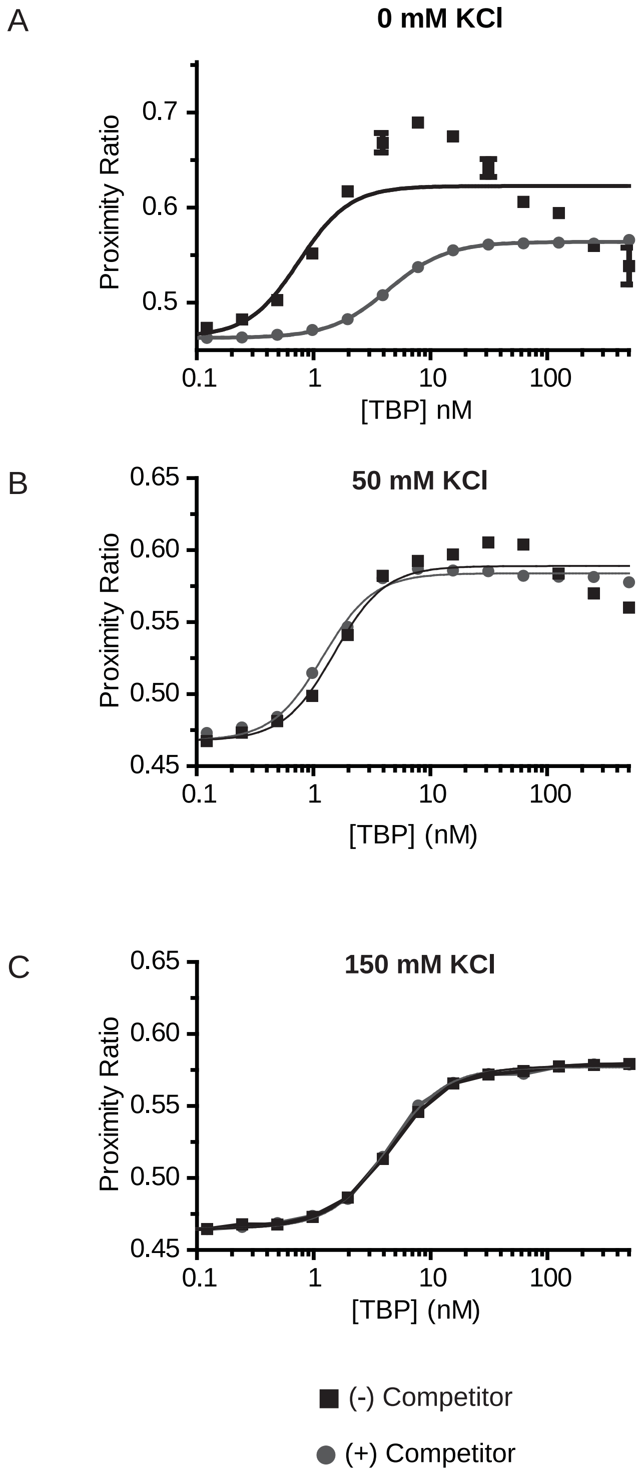

Figure S5. Non-specific contacts are the reason for assymmetric binding curves. (A-C) Binding curves showing TBP binding to TATA-14I DNA at 0 (A), 50 (B), and 150 (C) mM KCl in the absence (-) or presence (+) of unlabeled 35 bp competitor DNA co-titrated in equal molar concentration with TBP. Upon binding, proximity ratio decreases at eleveated TBP concentrations in the absence of competitor, but in the presence of competitor, the curve reaches a saturatable plateau. These data show at high ionic strength that non-specific TBP association is highly subdued and the addition of competitor adds no extra benefit. The sequence of the double-stranded competitor DNA is GGTGCCGAGGCCGTCAATTGCTCG-TAGACAGC, containing no apparent TATA-box sequence. Data are fit to the hill equation for single-site binding.

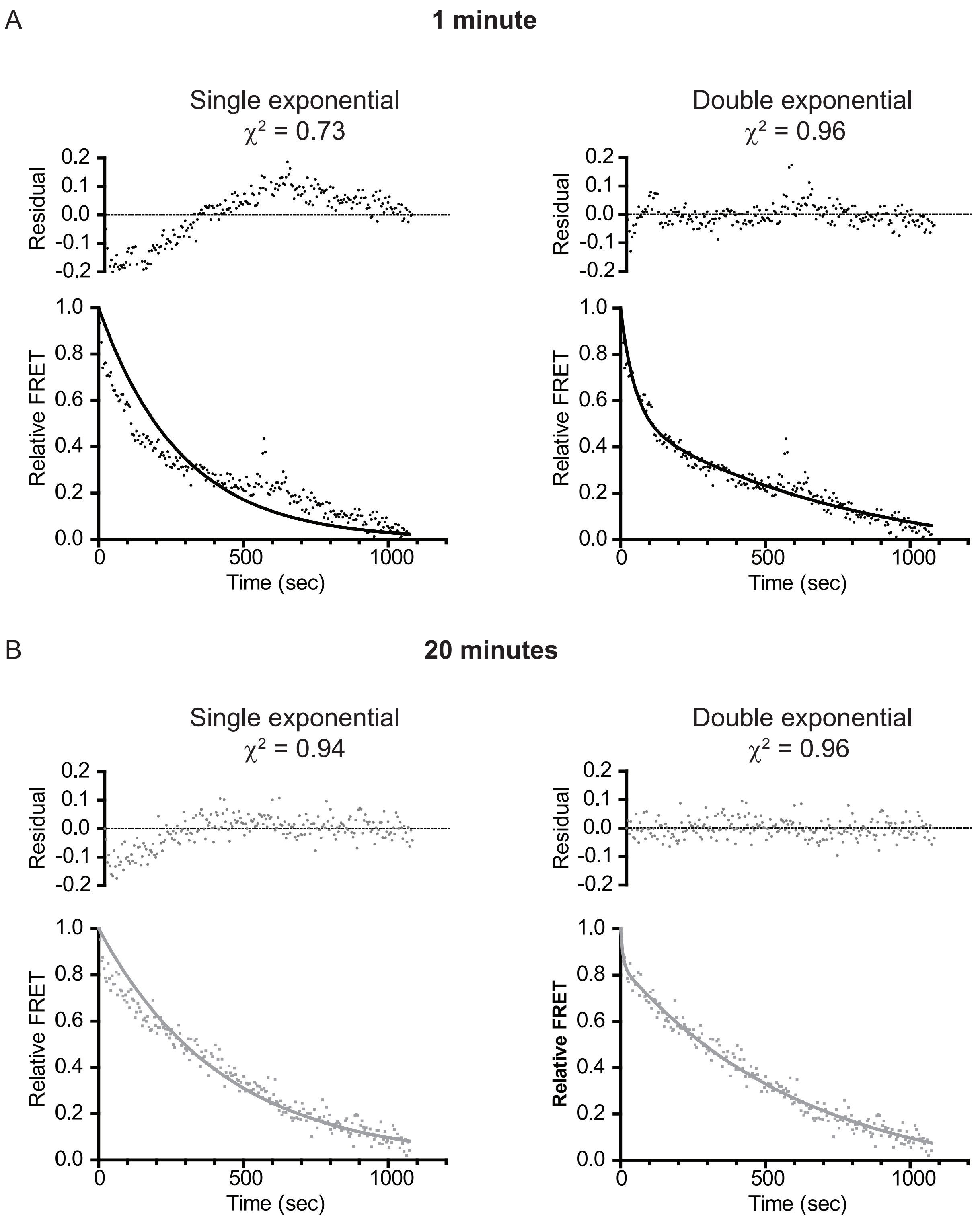

Figure S6. Comparative analysis of TBP kinetic dissociation. A) A comparison of data for the 1 minute time-point shown in Fig. 4A fit to either a single or double exponential decay. Residuals are shown for each curve-fit relative to the data are shown above. Residuals highlight the deviation from a single-exponential after short incubation times, indicative of a heterogenious populaiton of molecules. B) Same as in (A) for the 20 minute timepoint. Residuals show comparable results for both single and double exponential, indicating a large majority of the population is in a uniform state.

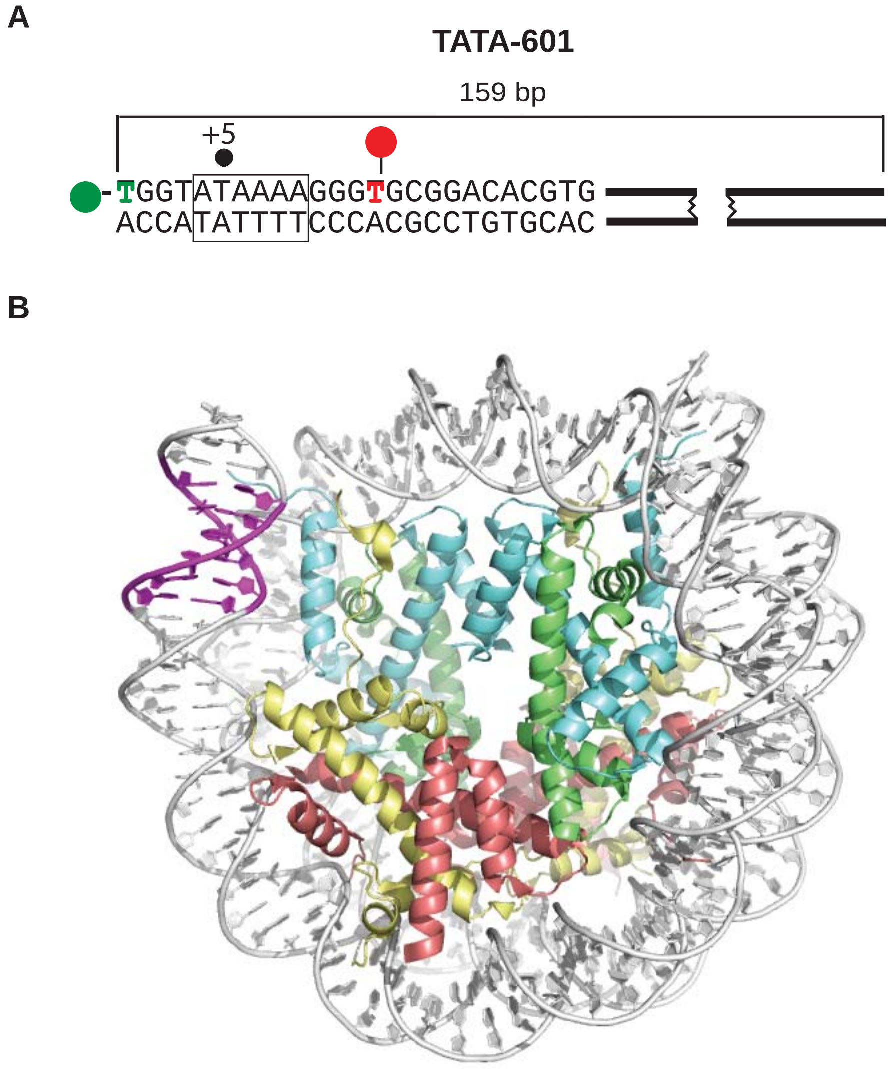

Figure S7. Construct used to test TBP access to nucleosomal DNA A) The 601 positioning sequence was used as a template for TBP binding, where a TATA-box was inserted approximately +5 bp from the last contact point with the nucleosome. The DNA was dual labeled on the end with donor and internally 14 bp away with acceptor fluorophores. An additional 10 bp of DNA flanks the linker arm opposite of the TATA-box. **B)** A schematic showing the location of the TATA-box based upon the crystal structure of a nucleosome containing 601 DNA from pdb accession code 3LZ0 (65). As can be seen, the minor groove of the TATA-box (magenta) spans the region of first contacts with the nucleosome near the histone H3 N-terminal α-helix. Shown are H2A (yellow), H2B (red), H3 (cyan), H4 (green), and DNA (grey).

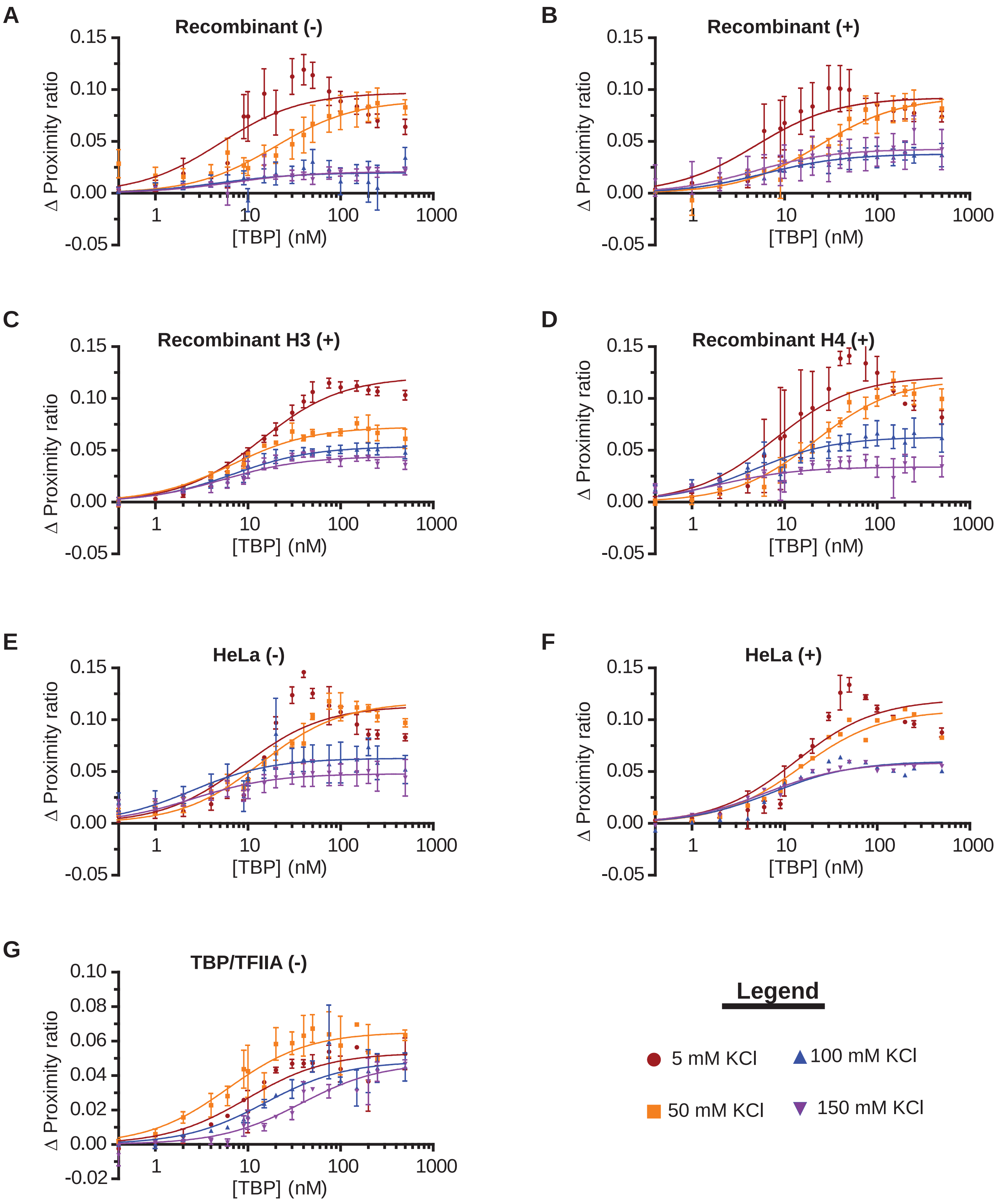

Figure S8. Data from globally plotting and fitting TBP binding to nucleosomes. (A-G) Shown are curves for TBP binding to 601-TATA nucleosomes under different salt and acetylation conditions; (-) and (+) indicate nonacetylated and hyper-acetylated histones, respectively. Unless indicated, histone octamer was acetylated. Data points are averages and error bars represent one standard deviation or a range of two values.

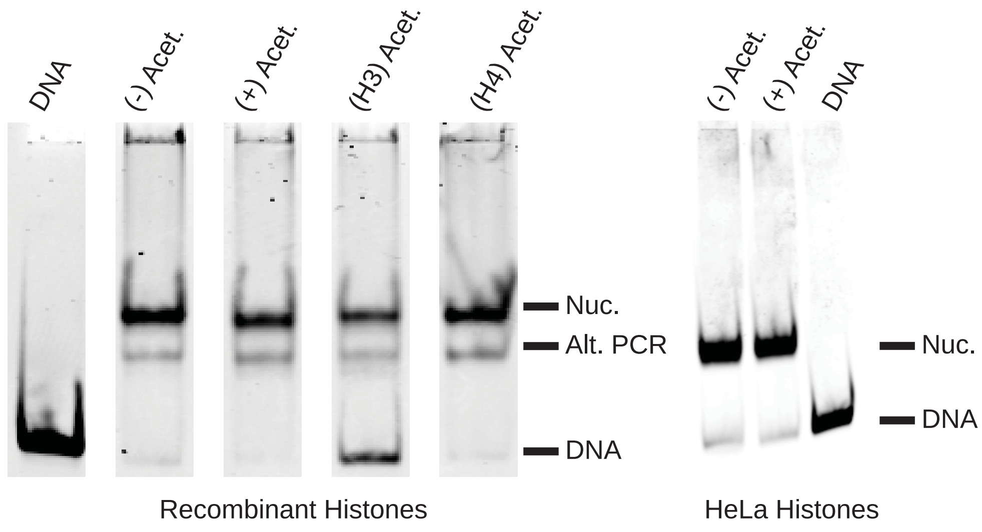

Figure S9. Native PAGE gels showing nucleosome preparations used for study. Samples were incubated in RB and loaded onto a 5% native page 0.5X TBE and run at 300 V for 3 hrs. Gels were fluorescently imaged using a typhoon imager with settings for the Atto 633 dye. Samples show high quality with little unassociated DNA and alternative nucleosome species. Abbreviations: Nuc. - Nucleosome, Alt. PCR. - Alternative PCR product.

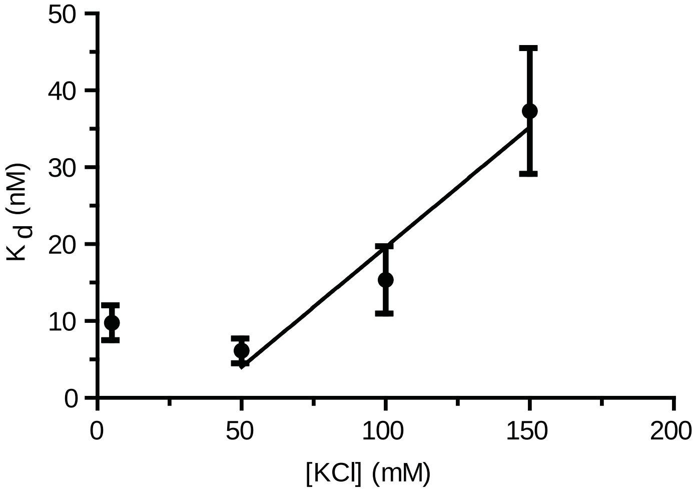

Figure S10. A plot showing the salt-dependent apparent binding affinity of TBP and TFIIA to TATA-601 nucleosomes. Specific TATA-box binding is repressed at elevated salt concentrations. Error bars represent the range of two independent binding experiments.

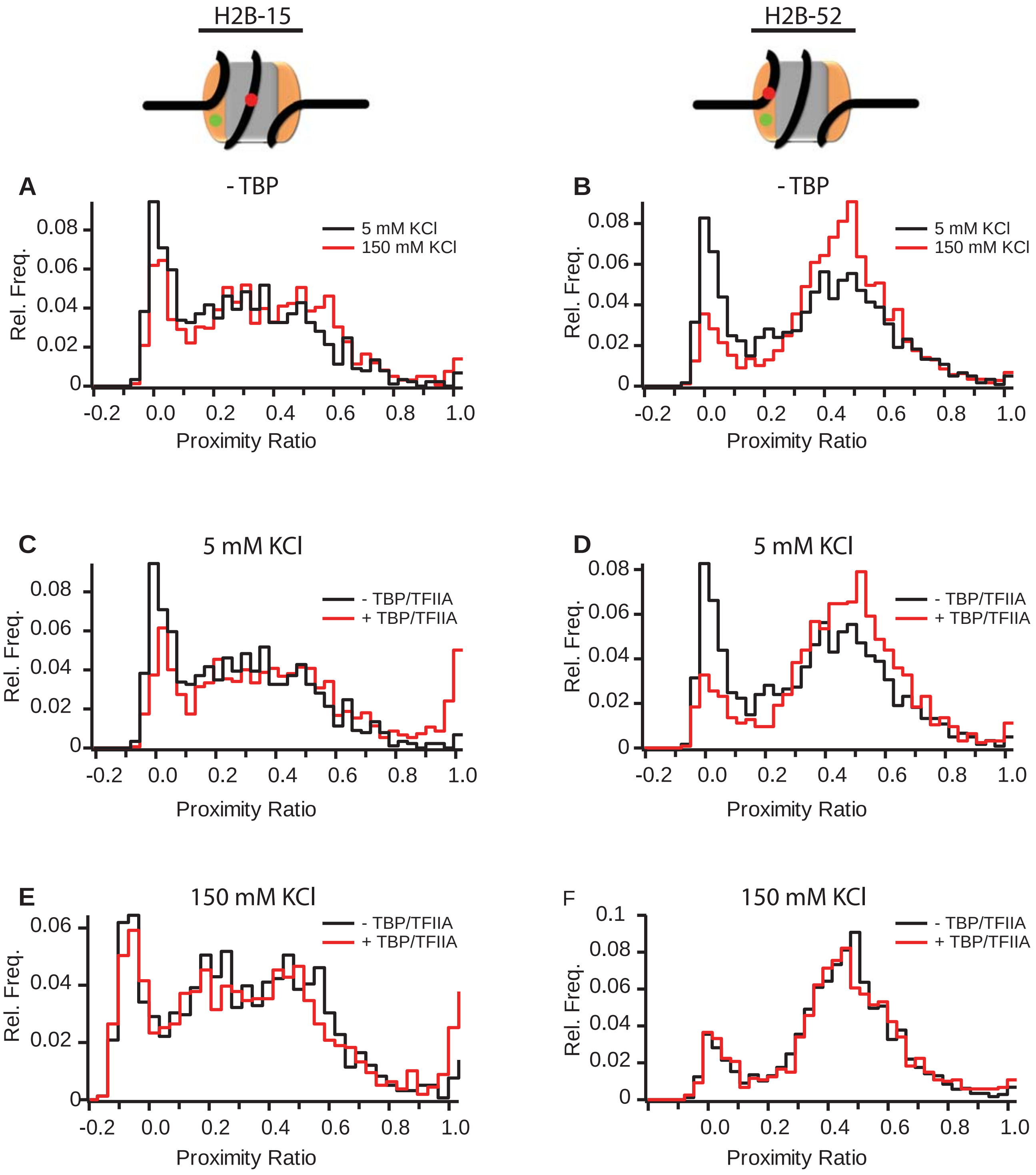

Figure S11. Nucleosome conformation is independent of TBP binding. Nucleosomes were constructed which contained FRET pairs on H2B and positions 15 bp (A,C,E) and 52 bp (B,D,F) from the nucleosome dyad. DNA contained the TATA-box in the identical site as TATA-601 DNA. **(A-B)** spFRET was measured and histograms generated under 5 mM or 150 mM KCl in the absence of TBP and TFIIA for H2B-15 and H2B-52, respectively. **(C-F)** spFRET measured on H2B-15 and H2B-52 in the absence or presence of TBP and TFIIA at 5 mM KCl (C,D) and 150 mM KCl (E,F).
